# Application of Bar Adsorptive Microextraction for the Determination of Levels of Tricyclic Antidepressants in Urine Samples

**DOI:** 10.3390/molecules26113101

**Published:** 2021-05-22

**Authors:** Mariana N. Oliveira, Oriana C. Gonçalves, Samir M. Ahmad, Jaderson K. Schneider, Laiza C. Krause, Nuno R. Neng, Elina B. Caramão, José M. F. Nogueira

**Affiliations:** 1 Centro de Química Estrutural, Faculdade de Ciências, Universidade de Lisboa, Campo Grande, 1749-016 Lisboa, Portugal; mariananetoliveira@hotmail.com (M.N.O.); ocgp98@gmail.com (O.C.G.); samir.marcos.ahmad@gmail.com (S.M.A.); 2 Molecular Pathology and Forensic Biochemistry Laboratory, Centro de Investigação Interdisciplinar Egas Moniz (CiiEM), Instituto Universitário Egas Moniz (IUEM), Campus Universitário—Quinta da Granja, Monte da Caparica, 2829-511 Caparica, Portugal; 3 Forensic and Psychological Sciences Laboratory Egas Moniz, Campus Universitário—Quinta da Granja, Monte da Caparica, 2829-511 Caparica, Portugal; 4 Instituto de Química, Universidade Federal do Rio Grande do Sul, 91509-900 Porto Alegre, Brazil; jadersonqmc@gmail.com (J.K.S.); laiza_canielas@hotmail.com (L.C.K.); elina@ufrgs.br (E.B.C.); 5 Departamento de Química e Bioquímica, Faculdade de Ciências, Universidade de Lisboa, Campo Grande, 1749-016 Lisboa, Portugal; 6 Programa de Pós-Graduação em Biotecnologia Industrial, Universidade Tiradentes, 49032-490 Aracaju, Brazil

**Keywords:** tricyclic antidepressants, urine samples, bar adsorptive microextraction (BAμE), novel sorbent phases, biomaterials waste, flotation sampling technology, GC-MS

## Abstract

This work entailed the development, optimization, validation, and application of a novel analytical approach, using the bar adsorptive microextraction technique (BAμE), for the determination of the six most common tricyclic antidepressants (TCAs; amitriptyline, mianserin, trimipramine, imipramine, mirtazapine and dosulepin) in urine matrices. To achieve this goal, we employed, for the first time, new generation microextraction devices coated with convenient sorbent phases, polymers and novel activated carbons prepared from biomaterial waste, in combination with large-volume-injection gas chromatography-mass spectrometry operating in selected-ion monitoring mode (LVI-GC-MS(SIM)). Preliminary assays on sorbent coatings, showed that the polymeric phases present a much more effective performance, as the tested biosorbents exhibited low efficiency for application in microextraction techniques. By using BAμE coated with C_18_ polymer, under optimized experimental conditions, the detection limits achieved for the six TCAs ranged from 0.2 to 1.6 μg L^−1^ and, weighted linear regressions resulted in remarkable linearity (*r*^2^ > 0.9960) between 10.0 and 1000.0 μg L^−1^. The developed analytical methodology (BAμE(C18)/LVI-GC-MS(SIM)) provided suitable matrix effects (90.2–112.9%, RSD ≤ 13.9%), high recovery yields (92.3–111.5%, RSD ≤ 12.3%) and a remarkable overall process efficiency (ranging from 84.9% to 124.3%, RSD ≤ 13.9%). The developed and validated methodology was successfully applied for screening the six TCAs in real urine matrices. The proposed analytical methodology proved to be an eco-user-friendly approach to monitor trace levels of TCAs in complex urine matrices and an outstanding analytical alternative in comparison with other microextraction-based techniques.

## 1. Introduction

According to the World Health Organization, depression is a common mental disorder, being one of the biggest causes of incapacity around the world. Depression can be characterized by a vast number of symptoms, including but not limited to sadness, low self-esteem, difficulty to sleep, loss of appetite, fatigue, low concentration, and poor decision making. In the most severe cases it can even lead to suicide. Antidepressants are an effective way of treatment, which allows the patients to live a normal life. The first commercially available antidepressant, introduced in 1955, was imipramine, a tricyclic antidepressant (TCA) still widely used today [1,2,3]. However, these compounds have narrow therapeutic range (between 50 and 300 µg L^−1^ in plasma). When concentration exceeds 500 µg L^−1^, toxic effects, such as high body temperature, sleepiness, confusion, cardiac arrest, among others, can happen and, when it rises to 1000 µg L^−1^, death may also occur. Therefore, it is essential to develop sensible, accurate and simple analytical methods together with suitable sample preparation approaches for the determination of these pharmaceutical compounds in forensic matrices [4,5,6]. Nevertheless, when dealing with very complex matrices like biological matrices, a sample preparation step is always a must. In the past decades there has been a concern in making sample preparation more eco-friendly, leading to new strategies that include several innovative concepts, namely, miniaturization, simplification, much higher selectivity and sensitivity, the elimination of toxic organic solvents, and reduction the sample amount. Apart from other microextraction approaches such as solid-phase microextraction (SPME) or stir bar sorptive extraction (SBSE) [7,8,9,10,11,12,13], bar adsorptive microextraction (BAμE), which was introduced in the last decade, presents several advantages and has shown great simplicity and versatility by allowing the choice of sorbent coating for each particular type of application. Materials such as activated carbons (ACs) prepared from several sources, alumina, silica, cork, polystyrene divinylbenzene (PS-DVB), modified pyrrolidine, silica-based polymers, and nanomaterials, such as carbon nanotubes, and multi-walled carbon nanotubes have been used, demonstrating great performance as sorbent phases for BAμE technique [14,15,16,17,18,19]. Nevertheless, the preparation and application of new materials with specific sorption characteristics is still very important for particular applications, especially those from ecological sources, such as biomaterial waste. In addition, the BAμE devices can be lab-made, the experimental procedure is easy to implement and cost-effective, requires low sample volume and negligible amounts (100 µL) of organic solvent during the back-extraction step, presents remarkable performance, high enrichment factors and analytical limits at the trace level. Recently, new generation BAμE devices were introduced aiming to improve the overall procedure, promoting a better interfacing with the instrumental systems, as well as an alternative option for the routine work. The novel devices are smaller and more flexible, prepared with cylindrical nylon supports coated with suitable adhesive films where the sorbents are fixed [10,14,20,21,22,23].

**Figure 1:**
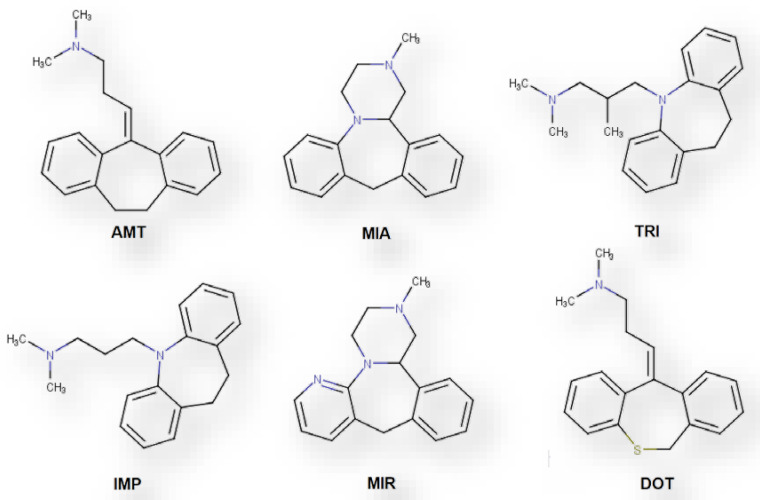
Chemical structures of the six TCAs studied in the present work.

In the present work, a new analytical strategy is proposed for trace determination of amitriptyline (AMT), mianserin (MIA), trimipramine (TRI), imipramine (IMP), mirtazapine (MIR) and dothiepin (DOT) (Figure 1) in urine matrices, using state-of-the-art BAµE devices, coated with several phases, in combination with large-volume-injection gas chromatography-mass spectrometry operating in the selected-ion monitoring mode (LVI-GC-MS(SIM)). It is also our goal to test, compare and discuss the selectivity and performance of several novel sorbents, having particular characteristics, prepared from biomaterials waste. The development, optimization, validation, and application in urine samples is also addressed.

## 2. Results and Discussion

### 2.1. LVI-GC-MS(SIM) Optimization

The initial step of this work was establishing the instrumental conditions that better fit the compounds under study. A mix solution of six TCAs was analyzed by GC-MS operating in the full-scan mode acquisition, which allowed to obtain the target ions (base peaks) and quantifier ions, also in accordance with the literature (Table 1) [24,25,26,27]. The obtained chromatogram by LVI-GC-MS(SIM) showed symmetrical peak shapes in less than 20 min of running time. The instrumental analytical thresholds were evaluated through the LODs and LOQs, corresponding to S/N of 3:1 and 10:1, in which 0.50 μg L^−1^ and 1.65 μg L^−1^ were achieved, respectively. The instrumental linearity was also assessed with eleven concentration levels ranging from 2.4 to 2500.0 μg L^−1^. Linear regressions showed plots with good linearity with determination coefficients (*r*^2^) higher than 0.9958.

### 2.2. Optimization of the BAµE-µLD Efficiency

The first task in the BAµE optimization process is the selection of the most suitable sorbent phase for the target analytes involved. Afterwards, several experimental parameters were studied using one-variable-at-a-time (OVAT) strategy, both for microextraction and back-extraction stages, including the desorption solvent and time, stirring rate, matrix pH, organic modifier, ionic strength, equilibrium time, and sample dilution effect, according to previous reports [8,23,28,29,30,31,32,33,34,35,36]. Although OVAT does not allow the identification of possible interactions between variables and requires many assays, this strategy is easy to implement in this type of optimizations since the number of variables is low and no relevant interactions are expected in compliance with our expertise [8,37].

#### 2.2.1. Selection of Sorbent Coatings and Back-Extraction Conditions

As usual in the BAµE technique, we started by selecting the best sorbent for the six TCAs and, for the present study, we proposed to test new and conventional sorbents. Thus, four new ACs (AC1, AC2, AC3 and AC4) prepared from biomaterials waste, as well as six commercial polymers (SX, HLB, C18, SDVB, SCN and DVBM) were assayed under the following experimental conditions; extraction stage: 5 mL of ultrapure water spiked with 100 µL of TCAs mix solution (500.0 µg L^−1^), pH 5.5, 3 h (990 rpm); back-extraction stage: 90 µL of MeOH, 30 min under ultrasonic treatment (42 ± 2.5 kHz, 100 W).

**Figure 2:**
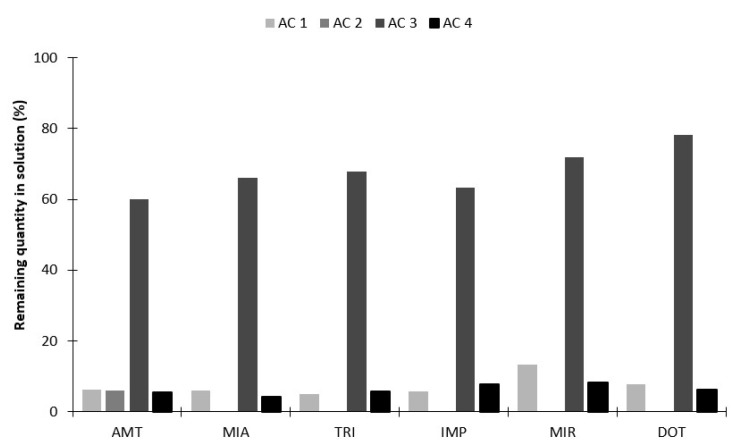
Remaining TCAs present in the aqueous matrix after the microextraction stage using the four different ACs prepared from biomaterials waste.

In a first approach, it was evaluated the adsorptive properties of the four new ACs prepared from coconut fiber (AC1), coffee residue (AC2), sugarcane chaff (AC3) and sugarcane bagasse (AC4) wastes, for the six target analytes. These biomaterials have already been successfully tested to remove metal ions from aqueous solutions [38] and proven to be ideal sorbents in environmental applications, namely for water cleaning treatment. Preliminary assays from these new AC phases showed that the recoveries yields were maximized at 12% for the six target TCAs (data not shown). Although these biomaterials have shown remarkable performance for adsorption of metal ions from aqueous media, based on the exploratory data obtained for organic compounds, we postulated that the observed lack of efficiency can be attributed to the back-extraction stage, despite the highly probable efficiency of the previous microextraction stage. Therefore, to evaluate this hypothesis, additional experiments were carried out to verify the ability of the ACs to extract the six TCAs from the aqueous medium. For this purpose, we used similar conditions for microextraction, although assaying a solution of ultrapure water (5 mL) plus 25 µL of the TCAs mix solution (100.0 mg L^−1^) and 16 h of equilibration time. Subsequently, a portion of the aqueous sample resulted from the microextraction stage was removed and extracted with dichloromethane (50:50, *v*:*v*), followed by ultrasonic treatment (42 ± 2.5 kHz, 100 W, 5 min) and analysis of the organic phase by LVI-GC-MS(SIM). The results obtained and depicted in Figure 2 shows, apart from the AC3 sorbent, that all the remaining carbon-based coatings (AC1, AC2 and AC4) presented remarkable microextraction efficiencies, since the resulting aqueous media do not present levels of TCAs higher than 15%. In this sense, we found that the proposed back-extraction stage conditions would not be able to efficiently remove the adsorbed TCAs, due to the very strong interactions established with the surface of these novel biosorbents, leading to low overall recovery yields. It should be emphasized that the analytical process of the BAμE technique is always characterized by a two-stage balancing process (adsorption-desorption equilibrium), in which the adsorption phenomena must be sufficiently effective during the microextraction stage, but not too strong that could compromise the effectiveness of the subsequent back-extraction stage. Like this, from the preliminary data achieved, we can deduce that the four biosorbents tested herein seem very effective from the adsorption point of view, ideal for removal processes (e.g., water decontamination, etc.), yet presenting great limitations as coating phases for microextraction-based techniques.

**Figure 3:**
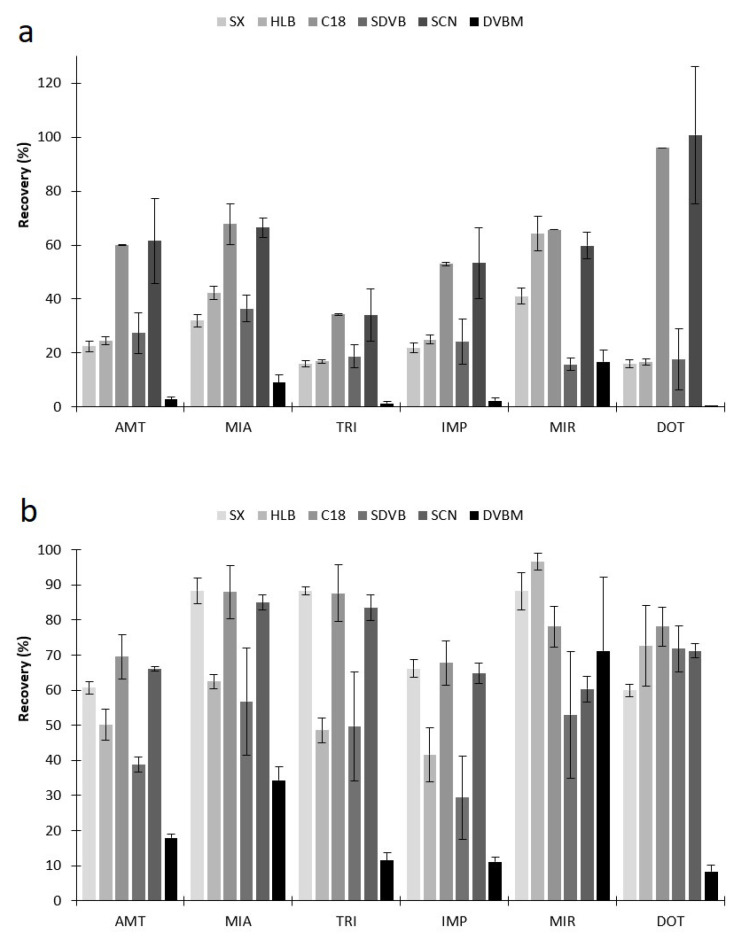
Effect of MeOH (**a**) and MeOH:ACN mix (**b**) stripping solvents on the back-extraction of the six TCAs from the different polymer phases by BAµE-µLD/LVI-GC-MS(SIM). The error bars represent the standard deviation of three replicates.

This finding may also be associated due to the large surface area and small pore size (<20 Å) presented by the four ACs under study, which may promote strong interactions with the target compounds and may hinder the back-extraction stage. For this reason, we decided to discard these biomaterial sorbents and assayed the six commercial polymeric phases (SX, HLB, C18, SDVB, SCN and DVBM), since they seem to be more suitable for the chemical structures of the target molecules involved. Figure 3 present assays performed by using these six polymer-based coatings, showing the efficiency achieved for the six TCAs, under similar experimental conditions, where the best selectivity is obtained for C18 and SCN phases. Although these polymers are structurally very different, the results obtained were expected, once the C_18_ polymer has a long aliphatic chain promoting strong hydrophobic interactions with the aliphatic chains present in the molecular structures of the target TCAs. On the other hand, the SCN polymer can promote dipole-dipole interactions through the cyano group and the electronegative elements (N and/or S) present in the chemical moieties of the six TCAs. Even so, we decided to evaluate the influence of the stripping solvent involved during the desorption step for all six polymer-based coatings. In general, the most common solvents used for the back-extraction stage in BAμE technique are MeOH (Figure 3a), ACN and mixtures of both (Figure 3b) [8,18]. In this sense, the data achieved showed that the use of an equivalent volume of MeOH and ACN presented the best performance, which allowed the selection of three polymeric phases, i.e., SX, C18 and SCN, the former promoting interactions through the N-vinylpyrrolidone group, demonstrating the great influence of the solvent involved during the back-extraction stage.

Even so, to speed up the back-extraction process, ultrasonic treatment was also implemented by using 15, 30, and 45 min of sonification time. The results obtained (data not shown) lead us to quit using the SCN phase once it showed the worst efficiency among the other two sorbent coatings. Therefore, the SX and C18 sorbents were selected for further assays and it has been found that at least 15 and 30 min are respectively needed to fully desorb the six TCAs from the microextraction devices.

#### 2.2.2. Microextraction Parameters

In this section, the first parameter optimized was the effect of the agitation stirring, in which three different speeds were tested, i.e., 750, 990, and 1250 rpm. From the results achieved (data not shown), it was observed that the best results occurred when 990 rpm are used, in line with previous reports [29,30,31,32,33,34,35,36] and having been selected for further assays.

**Figure 4:**
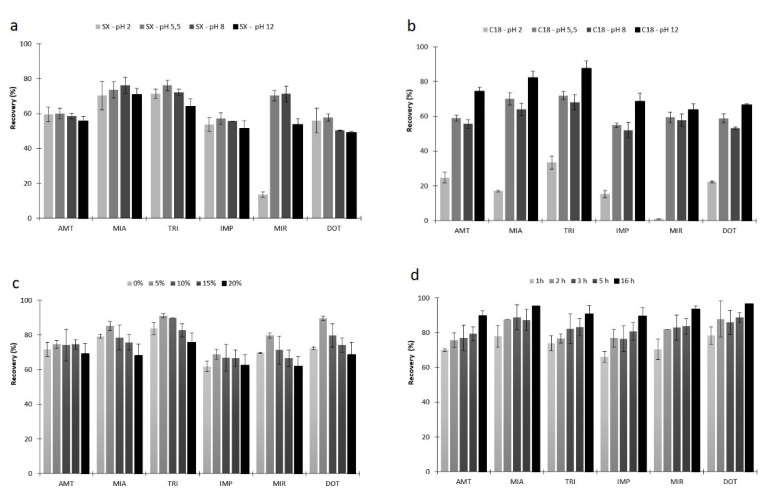
Effect of matrix pH using SX (**a**) and C_18_ (**b**) sorbent phases, percentage of NaCl (**c**) and equilibrium time (**d**) on the microextraction efficiency of six TCAs in aqueous media by BAµE-µLD/LVI-GC-MS(SIM). The error bars represent the standard deviation of three replicates.

Afterwards, the solution pH was also adjusted in order to promote partially or completely non-ionized TCAs, which may condition the the recovery yields through reverse-phase interactions [8,29,39]. The p*K*_a_ values for the six TCAs under study vary between 6 and 10, noticing that at pH 12, all of them are in the non-ionized form. Figure 4a,b show the results obtained from assays performed at several pH values (2.0, 5.5, 8.0 and 12.0), where the best data are attained by using the C_18_ coating at pH 12.0, once the six target TCAs become non-ionized [40]. However, when using the SX phase, the recovery yields did not vary significantly with pH variation. This observation can be attributed to additional chemical interactions promoted by the latter sorbent phase, other than reverse-phase type, e.g., dipole-dipole. For this reason, the optimization was pursued using only C_18_ polymer at pH 12.

The next optimization step was to study the effect of the medium polarity on the recovery yields for the six TCAs, varying the content (0 to 20%) of an organic modifier (MeOH). It is usually employed to minimize the possible adsorption of the analytes to the glass walls of the sampling flask (“wall-effect”), which generally occurs for compounds with log *K*_O/W_ higher than 4. However, it can also lead to a solubility increment of the analytes in the aqueous media, hindering the microextraction process [8,28]. As expected, and once the six TCAs present log *K*_O/W_ lower than 5, the best results were attained without MeOH addition.

Afterwards, the matrix ionic strength was evaluated by increasing amounts of NaCl (0 to 20%). An inert salt is usually added to the aqueous matrix to promote the “salting-out” effect, reducing the target compounds solubility in water. Commonly, for analytes presenting a log *K*_O/W_ higher than 3.5, the addition of an inert salt does not improve the recovery yields through the BAμE approach. This can be caused by several reasons; it can caused by the “oil-effect” phenomena, promoting the migration of non-polar compounds to the surface of the aqueous media, minimizing the contact between the analytes and the microextraction device; it can enhance the viscosity by decreasing the equilibrium kinetics; and the polymeric surface area can be blocked through the salt ions. Nevertheless, discrepancies are sometimes observed, and therefore, this parameter should be always evaluated [8,28]. Figure 4c depicts the obtained results, where the highest recovery yields were obtained by using 5% of NaCl. A possible explanation to this finding is that there is a maximum efficiency until that value, where the “salting-out” effect takes place, i.e., the available water to dissolve the analytes is reduced with the consequent increment on the recovery yields. However, above 5% of NaCl, the “salting-in” effect can also occur, where the analytes can interact with the salt ions through electrostatic interactions and decreasing the recovery, in agreement with previous reports [41].

The equilibrium time was the next parameter to be optimized, since in equilibrium conditions the maximum sensitivity and precision are achieved [8]. In Figure 4d, it is possible to observe that the best results are achieved when the equilibrium time lasts for 16 h, in agreement with previous reports, where a comparable behavior was observed [23]. Even so, despite the substantial time involved, the analytical process can be performed overnight without any special requirement. It is noteworthy that 16 h are needed to achieve maximum recovery yields, although we can greatly reduce the equilibrium time and still obtain efficiencies of about 65% by using only 1 h.

Finally, the effect of the sample dilution was also studied and, as expected, by keeping the amount of the sorbent phase fixed and decreasing the sample volume, the recovery yields increase due to the increase in the concentration factor. Thus, with a larger sample volume, an instrumental response should increase due to the greater enrichment of the extracted analytes, with the consequence of an increase in the equilibrium time [8,42,43,44]. In this sense, the variation of the sample volume was evaluated for five different levels (1.5, 5.0, 10.0, 25.0 and 40.0 mL), with the results (data not shown) showing that the best response was obtained with the use of only 5 mL of aqueous solution.

**Figure 5:**
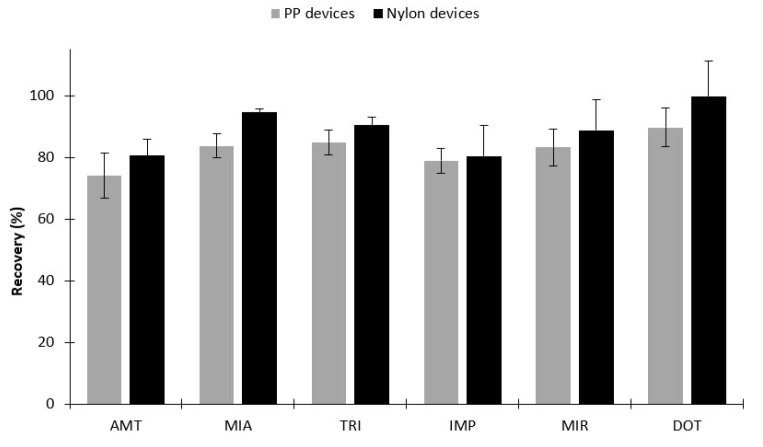
Effect of substrate type on the microextraction efficiency for the six TCAs in aqueous media by BAµE(C18)-µLD/LVI-GC-MS(SIM). The error bars represent the standard deviation of three replicates.

From the beginning, the BAµE devices used in this work were prepared with PP subtracts. Nevertheless, to make them more compatible with the instrumental systems, as well as to improve the routine work, new generation devices were already proposed using nylon subtracts [23]. They are much smaller, more flexible, user-friendly, allows the back-extraction stage in an only single step and compatible with the injection operation of the conventional instrumental systems. So far, these devices had only been combined with HPLC systems. In this work, we decided to combine, for the first time, the new generation BAµE devices with a GC-MS system. For this purpose we decided to compare the performance of the conventional devices with the new generation ones, as depicted in Figure 5. From the data obtained, very similar performances are achieved between both devices. Therefore, given the advantages of the nylon subtracts, these were adopted for the validation and application sections.

At the end of the optimization procedure, the attained recovery yields were between 80.5 and 99.6% for the six target TCAs (RSD < 12.1%), under the following experimental conditions; extraction stage: 5 mL of ultrapure water spiked with 100 µL of TCAs mix solution (500.0 µg L^−1^), pH 12, 5% NaCl, 3 h (990 rpm); back-extraction stage: 90 µL MeOH/ACN (50:50), 30 min under ultrasonic treatment (42 ± 2.5 kHz, 100 W).

### 2.3. Validation of the Proposed Methodology

After the optimization section, we proceeded to the validation of the proposed methodology (BAµE(C18)-µLD/LVI-GC-MS(SIM)) using blank urine matrices. In a first approach, the analytical thresholds were determined through the LODs where values in between 0.20 and 1.56 µg L^−1^, were achieved. The linearity range was assessed between 10.0 and 1000.0 µg L^−1^ (nine concentration levels). This range was chosen once it included the therapeutic, toxic and lethal concentration values for TCAs [45]. For the calibration plots, we started by plotting a conventional linear regression, which showed good linearity (*r*^2^ ≥ 0.9914), and according to the lack-of-fit test (at the confidence level 95%), it presented a good fit for the present study, i.e., the F_calc_ was always below the F_tab_. Nevertheless, by studying the data dispersion, the relative residues were too high (>15%), and it was clearly visible a tendency in the residue plots, which showed heterogeneous profiles. As a result, heteroscedasticity was observed for all TCA plots, which might be caused by the large range of the concentrations considered. For this reason, ordinary least-squares linear regression method was not fit for the attained data, since it results in large errors, in particular for the lower concentrations. Given the evidence of heteroscedasticity, a weighted linear regression method was adopted, once it is the simplest and most effective way to compensate the data dispersion observed, especially for the lower concentration levels. The quality of the fit of weighted regressions can be evaluated by calculating the sum of percentage relative error (%RE). The appropriate weighting factor can be calculated from the inverse of the variance. Nevertheless, this is impractical, once it requires several determinations for each calibration point and a new calibration plot must be done every time that the methodology is applied. Therefore, an empirical weight based on concentrations (variable x) and responses (variable y) were used. Six weighting factors were tested, 1/y^1/2^, 1/y, 1/y^2^, 1/x^1/2^, 1/x and 1/x^2^. The data achieved shows that a weighting factor of 1/y resulted in the lowest %RE across the whole range. The weighting factor should be used in the calculation of the regression equation parameters as described in previous works [45,46,47,48].

As stated before, the LLOQ was defined as the lowest concentration value in which it is possible to quantify any analyte with precision and accuracy, i.e., it is the concentration in which the RSD and %RE are lower than 20%. Table 2 summarizes the validation parameters, namely the LODs, LLOQs, calibration equations and the *r*^2^ values achieved.

As previously mentioned, accuracy and precision were calculated for inter and intra-day at four spiking levels, as summarized in Table 3. The intraday accuracy and precision values were between −12.0 and 14.2%, and ranging from 0.4 to 15.8%, respectively. The interday accuracy and precision ranged from −8.2 to 20.0%, and between 2.1 and 19.9%, respectively. These data show that the proposed analytical approach presented suitable levels of accuracy and precision for trace level analysis of TCAs in urine matrices.

Matrix effects, average recovery yields and process efficiency were also performed at two different concentration levels (25 and 750 µg L^−1^), as presented in Table 4. 

Matrix effects were found to be in between 90.2 and 112.9% (RSD < 14.4%). Results above 100% show ionic enrichment and below, ionic suppression; 100% is the ideal, although it is impossible to eliminate matrix effects [49,50,51]. The average recovery yields achieved were between 92.3 and 111.5% (RSD < 12.4%). Finally, the process efficiency ranged from 84.9 to 124.3% (RSD < 13.9%). These data proved that the developed methodology is well suited to determine trace levels of TCAs in urine matrices.

### 2.4. Application to Real Urine Samples

**Figure 6:**
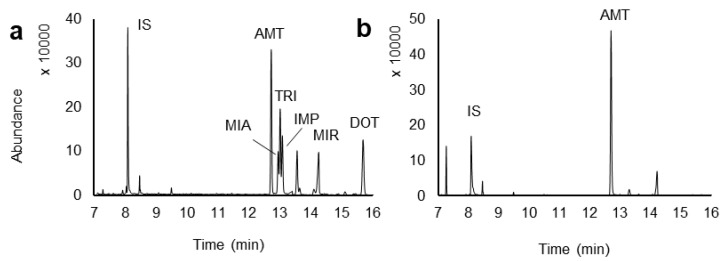
Chromatograms obtained from urine samples spiked at the 100.0 μg L^−1^ for six TCAs (**a**) and a positive anonymous donor without spiking (**b**) analyzed through BAµE(C18)-µLD/LVI-GC-MS(SIM) methodology, under optimized experimental conditions.

To test the applicability of the present methodology to real matrices, fifty-two urine samples from anonymous donors were analyzed, using GC-MS operating in the full-scan mode acquisition, to ensure possible positive identifications. Figure 6 depicts a chromatogram from a urine sample spiked at the 100.0 μg L^−1^ for the six TCAs (a) and a positive sample (b) for AMT with an amount of 158.87 ± 1.93 µg L^−1^, which is well above the therapeutic levels [52]. The figures of merit presented show that good selectivity and sensitivity are achieved by the proposed methodology.

### 2.5. Performance Comparison with Other Microextraction Techniques

In the present contribution, we decided to compare the developed methodology with other microextraction techniques dedicated for the analysis of TCAs in urine samples already reported in the literature [6,27,52,53,54,55], as summarized in Table 5. 

First, the obtained recovery yields and precision levels are much better or alike other analytical approaches, both in water and urine matrices. The attained analytical thresholds (LODs and LLOQs) compares favorably with the other proposed analytical methods. Unlike other methodologies already reported, the present work has the great advantage of encompassing therapeutic, toxic and lethal concentrations in its linear range. In short, the methodology proposed herein can be considered an alternative for the determination of the six TCAs involved in urine samples.

## 3. Materials and Methods

### 3.1. Chemicals and Standards

Methanol (MeOH, 99.9%), acetonitrile (ACN, 99.9%), formic acid (99%), acetic acid (99.5%) and dichloromethane (99.9%) were purchased from Carlo Erba (Barcelona, Spain). Sodium hydroxide (NaOH, 98.0%) was obtained from AnalaR BDH Chemicals (Leicestershire, UK). Phosphoric acid (85.0%) and disodium phosphate (99%) were purchased from Panreac (Barcelona, Spain). Diphenylamine (DIF, 98.0%) used as internal standard (IS) was purchased from Sigma-Aldrich (Saint Louis, MO, USA). Hydrochloric acid (HCl, 37%) was purchased from Sigma-Aldrich (Vienna, Austria). Ultra-pure water was obtained from Milli-Q water purification systems from Merck Millipore (Burlington, MA, USA). Six pharmaceutical tablets commercially available in the Portuguese market were used to prepare the standard solutions: ADT produced by Generis (Amadora, Portugal) containing 10 mg of amitriptyline hydrochloride (AMT); Tolvon produced by Merck Sharp & Dohme (Paço de Arcos, Portugal) containing 30 mg of mianserin hydrochloride (MIA); Surmontil produced by Laboratórios Vitória (Amadora, Portugal) containing 35 mg of trimipramine (TRI); Tofranil produced by Amdipharm (Dublin, Ireland) containing 10 mg of imipramine hydrochloride (IMP); Mirtazapina Alter produced by Alter (Alter do Chão, Portugal) containing 15 mg of mirtazapine (MIR); Protiadene produced by Teofarma (Pavia, Italy) containing 75 mg of dothiepin hydrochloride (DOT). Stock solution were prepared according to previously described protocols [56,57], where each tablet was individually meshed and dissolved in 10 mL of MeOH giving different concentrations according to the pill initial dosage. Then, the solutions were sonicated (42 ± 2.5 kHz, 100 W, Branson 3510, Switzerland) for 15 min, centrifuged at 3000 rpm for 10 min. Diphenylamine stock solutions were prepared by dissolving in MeOH to give a final concentration of 1000 mg L^−1^, stored at −20 °C in glass flasks and renewed every month.

### 3.2. Sorbent Phases

The sorbent phases used for coating the BAμE devices were from several ACs and polymers. The novel four ACs were prepared from biomaterials waste, characterized, and provided by the Industrial Biotechnology Laboratory from Tiradentes University (Aracaju, Sergipe, Brazil). AC1 was obtained from coconut fiber (pH_PZC_ 6.3, 1130 m^2^ g^−1^ (BET) surface area, <20 Å pore size); AC2 was obtained from coffee residue (pH_PZC_ 7.3, 1308 m^2^ g^−1^ (BET) surface area, <20 Å pore size); AC3 was obtained from sugarcane chaff (pH_PZC_ 5.9, 1185 m^2^ g^−1^ (BET) surface area, <20 Å pore size); and AC4 was obtained from sugarcane bagasse (pH_PZC_ 6.9, 791 m^2^ g^−1^ (BET) surface area, <20 Å pore size). The polymeric sorbents used were Strata-X (SX) (polymer containing N-vinylpyrrolidone; 33 μm particle size, 85 Å pore size, 800 m^2^ g^−1^ surface area), Strata-CN (SCN) (reversed phase polymer containing ciano; particle size 55 μm, 70 Å pore size, surface area 500 m^2^ g^−1^) and Strata SDB-L (SDVB) (reversed phase styrene-divinylbenzene polymer; particle size 100 μm, 260 Å pore size, 500 m^2^ g^−1^ surface area) from Phenomenex (Torrance, CA, USA); Oasis HLB (HLB) (reversed phase N-vinylpyrrolidone-divinylbenzene co-polymer; 30 μm particle size, 80 Å pore size, 830 m^2^ g^−1^ surface area and pH stability at 0–14) from Waters (Milford, MA, USA); ENVI-18 (C18) (reversed phase octadecyl silica polymer; 45 μm particle size, 60 Å pore size, 475 m^2^ g^−1^ surface area) from Supelco (Darmstadt, Germany); LiChrolut EN (DVBM) (reversed phase ethylvinylbenzene-divinylbenzene co-polymer; particle size 40–120 μm, 60 Å pore size, surface area 1200 m^2^ g^−1^) from Merck Millipore (Darmstadt, Germany).

### 3.3. Urine Matrices

Urine samples were obtained from the Joaquim Chaves Saúde clinic (Algés, Portugal) and provided in total anonymity without any information from the donors. Additional urine control samples were provided for the validation process from healthy volunteers who guaranteed not to have consumed any of the TCAs under study. Upon arrival at the laboratory, the samples were frozen (−20 °C) until use.

### 3.4. Experimental Set-Up

#### 3.4.1. Preparation of the BAμE Devices

The BAμE devices were lab-made prepared. For the optimization assays, the devices were made with polypropylene (PP) cylindrical subtracts, produced in similar way to previous works [30,31]. PP subtracts with 10 × 3 mm were coated with a suitable adhesive film, and then coated with powdered sorbents. To evaluate the similarity between the conventional used PP devices and the new generation of BAμE devices, nylon cylindrical subtracts were used. The nylon subtracts having 10 × 1 mm were coated with a suitable adhesive film, and then coated with powdered sorbents [23]. Before being used, and to remove potential impurities, the devices were cleaned with ultrapure water under magnetic stirring and dried in a clean Kimwipe [23,30,31].

#### 3.4.2. Optimization Assays

For the optimization assays, 5 mL of ultrapure water (pH 5.5) were added to a sampling glass flask and spiked with 100 μL of a mix solution of all six TCAs (500.0 μg L^−1^), resulting in a final concentration of 10.0 μg L^−1^. Afterwards, a conventional Teflon magnetic stirring bar and a BAμE device were introduced in the same flask. The microextraction process was performed through floating sampling technology using a multi-point agitation plate (Variomag HþP Labortechnik AG Multipoint 15, Oberschleissheim, Germany) at room temperature (25 °C). After microextraction, the BAμE devices were removed from the glass flasks with clean tweezers and a clean Kimwipe. For the back-extraction stage, the BAμE devices were placed into glass vial inserts having 90 μL of an organic solvent following by ultrasonic treatment (42 ± 2.5 kHz, 100 W) at room temperature (25 °C). Afterwards, 10 μL of a DIF solution (IS; 10,000.0 μg L^−1^) were introduced to the glass vial inserts, resulting in a final concentration of 1000.0 μg L^−1^. In the case of the PP devices and after the back-extraction stage, these are removed from the vial before being sealed and proceeding to the instrumental analysis. In the case of the nylon devices this step is not required. Blank assays without spiking were also performed. Unless specified, each assay was done in triplicate. Several parameters were studied to optimize the extraction efficiency following an OVAT strategy. In this approach all variables are fixed, except one, and the extraction efficiency is studied at several levels of this parameter. The selected parameters for the optimization process were desorption solvent (MeOH, ACN, and MeOH/ACN (50/50%, *v*/*v*) and time (15, 30, and 45 min), stirring speed (750, 990, and 1250 rpm), pH (2.0, 5.5, 8.0, and 12.0), organic modifier (MeOH: 0, 5, 10, 15, and 20%), matrix ionic strength (NaCl: 0, 5, 10, 15, and 20%), equilibrium time (1, 2, 3, 5, and 16 h) and sample dilution effect using different volumes (1.5, 5.0, 10.0, 25.0, and 40.0 mL), in accordance with previous reports [23,29,30,31,32,33,34,35,36].

#### 3.4.3. Pre-Treatment of Biological Samples

After thawing at room temperature (25 °C), the samples were subjected to an alkaline hydrolysis step to eliminate most of the interfering compounds from the urine samples, e.g., carbamide, uric acid, or calcium salts, etc. This process is similar to a previous described hydrolysis method [53]. In this case, 200 µL of NaOH 10 mol L^−1^ was added to 1 mL of urine and the solution was placed in an automatic evaporator (Laborota 4000 Efficient, Heidolph, Schwabach, Germany) at 60 °C for 10 min. Afterwards, the samples were centrifuged for 10 min at 2000 rpm, and the supernatant transferred to new clean vials and subjected to ultrasonic treatment (42 ± 2.5 kHz, 100 W) for 5 min. Lastly the samples were filtered (0.45 µm nylon filters, Laborspirit, Lisbon, Portugal).

#### 3.4.4. Validation and Real Sample Assays

Assays performed with urine samples were done after the optimal conditions were found. In this sense, for each assay, 1.2 mL of urine was added to 3.8 mL of buffer solution (pH 12.0, adjusted with HCl 5 mol L^−1^) with 5% of NaCl. For validation assays, 100 µL of TCAs mix solution was added, except when performing blank assays. For the validation process several parameters were evaluated, such as linearity, analytical thresholds, selectivity, accuracy, precision, recovery, matrix effects and the process efficiency, according to previous works [50,51]. To assess the developed methods selectivity, the optimized BAμE-μLD/LVI-GC-MS method was applied to urine control samples and the absence of interfering compounds at the studied TCAs retention time was verified. Calibration standards were prepared between 10.0 and 1000.0 μg L^−1^ to assess the linearity (estimated with the lack-of-fit test), the coefficients of determination (*r*^2^) and residuals dispersion. In order to determine intra and inter-day accuracy and precision, several assays (n = 6) were performed using different spiking concentrations of TCAs mix solution, including 10.0, 50.0, 500.0, and 1000.0 μg L^−1^, corresponding to the lower limit of quantification (LLOQ), low, medium, and high concentrations, respectively. Interday assays were performed in three consecutive days and intraday assays were performed in the same day. The acceptance criteria were that the relative residuals and relative standard deviations (RSDs) ≤ 15%, except for LLOQ values were ≤20% values were accepted. The analytical thresholds were assessed by the limit of detection (LOD) and LLOQ. The LOD corresponds to a signal-to-noise (S/N) ratio of 3/1, while LLOQ is the lowest concentration where it is possible to quantify according to accuracy and precision parameters, being the first point in calibration curve, and also it corresponds to a S/N < 10. Lastly, to obtain matrix effects, recovery yields, and process efficiency, three sets of samples, each at two different concentrations (25.0 and 750.0 μg L^−1^), were prepared. Set A samples consisted of a mix solution with all the TCAs at the previously stated concentrations. Set B samples were fortified after the microextraction and before liquid desorption. Set C samples were spiked before the microextraction. The ration between absolute peak areas of sets A and B allowed for the calculation of matrix effects, between B and C for the recovery yields calculation, and between A and C for the calculation of the process efficiency.

### 3.5. Instrumental Set-Up

GC-MS analyses were performed with an Agilent Technologies system (Santa Clara, CA, USA) constituted by an Agilent 6890 series gas chromatograph, equipped with an Agilent 7683 automating liquid sampler and a programmed temperature vaporization injector, coupled to an Agilent 5973N mass selective detector. All data recorded and instrumental control was performed in the MS ChemStation software (G1701; version E.02.02.1431). Injection was made in solvent vent mode (vent time: 0.3 min, 50.0 mL/min, pressure 0.0 psi, purge flux: 60.0 mL/min, time 2.0 min) with programmed temperature starting at 80 °C (0.45 min) to 280 °C (600 °C/min, 3 min isothermal). Large-volume-injections (LVI) of 10 µL at 100 µL/min were performed. A capillary column was used Zebron ZB-5 (30.0 m × 0.25 mm × 0.25 μm; 5% phenyl, 95% dimethylpolysiloxane) (Phenomenex) for the GC analysis, along with helium as the carrier gas, in constant pressure mode (9.82 psi). Oven temperature was programmed starting at 80 °C (held for 1 min), to 240 °C (20 °C/min, for 5 min), then to 245 °C (1 °C/min), and finally 300 °C (20 °C/min), achieving a total run time of approximately 22 min. The transfer line temperature was 280 °C, the quadrupole analyzer temperature was 150 °C, and the ion source temperature was 230 °C. A solvent delay of 7 min was selected. Electron ionization was used (70 eV) in a range of masses between 35 and 550 Da, in the full-scan mode, with an ionization current of 34.6 µA and a multiplier voltage of 1200 V. In the selected ion monitoring (SIM) mode several groups of ions were monitored in a defined time frame according to their retention time, maintaining a dwell time of 100 ms^−1^. All the instrumental data were performed in triplicate and the calculations of each assay were performed by comparing the average peak areas of the extracted compounds to the IS peak area.

## 4. Conclusions

The analytical methodology proposed in the present work was fully developed, optimized, validated and applied for the determination of trace levels of selected six TCAs in urine matrices. The results show that the proposed approach compares favorably with other analytical strategies already reported in the literature, especially regarding the recovery yields and linear range. The proposed method is simple and user-friendly, suitable for trace analysis of TCAs in urine samples, in compliance with green analytical chemistry principles and can be used as a tool for routine work.

## Figures and Tables

**Table 1:** Target ions (base peaks in bold) and quantifier ions for each TCA studied by LVI-GC-MS(SIM), under optimized instrumental conditions.

TCAs	Ions (*m*/*z*)	Retention Time (min)
**AMT**	**58**/202/215	12.91
**MIA**	**193**/220/264	13.13
**TRI**	**58**/249/294	13.22
**IMP**	58/**234**/280	13.28
**MIR**	**195**/208/265	13.77
**DOT**	**58**/202/295	15.96

**Table 2:** LODs, LLOQs, calibration equations and *r*^2^ achieved for the six TCAs through BAμE(C18)-μLD/LVI-GC-MS(SIM) methodology, under optimized experimental conditions.

TCAs	LODs (µg L^−1^)	LLOQs (µg L^−1^)	Calibration Equations	*r* ^2^
**AMT**	0.20	10.00	y = 34.4780 x − 0.0174	0.9974
**MIA**			y = 7.3701 x − 0.0026	0.9974
**TRI**			y = 13.0250 x − 0.0033	0.9988
**IMP**			y = 3.5938 x − 0.0027	0.9960
**MIR**	0.39		y = 11.2264 x − 0.0026	0.9982
**DOT**	1.56		y = 29.1992 x − 0.0094	0.9978

**Table 3:** Inter and intraday accuracy and precision levels obtained for the six TCAs at four different concentrations by BAμE(C18)-μLD/LVI-GC-MS(SIM) methodology, under optimized experimental conditions.

TCAs	Spiking Level (μg L^−1^)	Intraday			Interday		
		Accuracy (%) ± Precision (%)			Accuracy (%) ± Precision (%)		
**AMT**	10.0	5.1	±	8.2	20.0	±	15.0
	50.0	−5.5	±	4.6	−5.0	±	8.9
	500.0	1.7	±	4.8	2.9	±	12.2
	1000.0	4.8	±	8.8	−4.4	±	11.8
**MIA**	10.0	−6.6	±	4.3	−2.8	±	16.7
	50.0	−0.5	±	11.1	0.1	±	13.2
	500.0	4.8	±	8.6	1.9	±	11.8
	1000.0	−0.1	±	8.9	2.5	±	13.2
**TRI**	10.0	0.9	±	6.8	13.1	±	18.1
	50.0	−1.7	±	6.1	11.0	±	13.8
	500.0	5.7	±	4.8	13.0	±	9.2
	1000.0	−4.1	±	13.7	5.8	±	11.7
**IMP**	10.0	−8.4	±	9.0	14.8	±	17.6
	50.0	6.7	±	12.8	2.4	±	14.5
	500.0	−1.2	±	6.3	14.2	±	14.9
	1000.0	−0.5	±	11.2	−4.3	±	10.1
**MIR**	10.0	1.3	±	15.8	15.9	±	19.9
	50.0	13.3	±	0.9	14.5	±	2.1
	500.0	6.5	±	6.0	13.0	±	12.1
	1000.0	−0.7	±	7.3	11.1	±	13.8
**DOT**	10.0	8.1	±	0.4	14.1	±	9.4
	50.0	14.2	±	2.5	1.0	±	13.5
	500.0	−8.2	±	10.3	−4.5	±	14.5
	1000.0	−12.0	±	7.2	−8.2	±	10.1

**Table 4:** Matrix effects, recovery yields and process efficiency obtained for the six TCAs at two different concentrations by BAμE(C18)-μLD/LVI-GC-MS(SIM) methodology, under optimized experimental conditions.

TCAs	Spiking Level (μg L^−1^)	Matrix Effects (%) ± RSD (%)			Recovery Yields (%) ± RSD (%)			Process Efficiency (%) ± RSD (%)		
**AMT**	25.0	90.2	±	6.6	95.3	±	9.6	86.0	±	7.5
	750.0	93.7	±	12.5	107.9	±	6.9	101.1	±	12.9
**MIA**	25.0	100.1	±	9.8	95.3	±	7.2	95.4	±	8.6
	750.0	111.5	±	11.2	111.5	±	7.7	124.3	±	11.7
**TRI**	25.0	102.1	±	8.4	103.3	±	9.3	105.5	±	12.0
	750.0	99.3	±	8.2	109.0	±	5.0	108.2	±	7.9
**IMP**	25.0	91.9	±	4.1	92.3	±	11.1	84.9	±	10.4
	750.0	109.3	±	13.9	106.5	±	10.0	116.5	±	12.6
**MIR**	25.0	95.6	±	10.9	108.5	±	12.3	103.6	±	12.0
	750.0	112.9	±	14.4	99.0	±	9.9	111.7	±	13.9
**DOT**	25.0	91.3	±	4.4	99.6	±	8.9	90.9	±	8.4
	750.0	99.4	±	11.6	103.7	±	7.5	103.1	±	13.9

**Table 5:** Comparison between the present study and others analytical approaches already reported in the literature for each six TCAs determination in urine and water matrices.

Analytical Method	TCAs	Recovery Yields (%)	RSD (%)	LOD (µg L^−1^)	LOQ (µg L^−1^)	Linear Range (µg L^−1^)	*r* ^2^	Ref.
**BAμE-μLD/LVI-GC-MS(SIM)**	AMT, MIA, TRI, IMP, MIR, DOT	~100.0	<9.6	0.20	10.0 (LLOQ)	10.0–1000.0	0.9974	This study
**MSPE/HPLC-UV**	AMT, IMP	98.5–99.5 (RR)						[6]
**SPE/GC-MS**	AMT, MIA, TRI, MIR, DOT	64.4–99.8	6.0–20.6	1.0–2.5	-	1.0–320.0	0.9963–0.9996	[27]
**SI-HLLE-DSPE-DLLME-SFO/HPLC-UV**	AMT, IMP	69.0–84.0	3.0–4.0	0.2–0.3	0.7–1.1 (LOQ)	0.7–1000.0	0.9960–0.9970	[52]
**DLLME/HPLC-UV**	TRI	112.0	6.1	0.6	-	2.0–100.0	0.9946	[53]
**HF-LPME/HPLC-UV**	AMT, IMP,	-	6.8		-	-	-	[54]
**DLLME/GC-MS**	AMT, IMP	88.2–103.6	7.4–7.9	0.5	2.0	2.0–100.0	0.9990	[55]

DLLME: Dispersive liquid-liquid microextraction GC: Gas chromatography; HF-LPME: Hollow fiber-liquid phase microextraction; HPLC: High performance liquid chromatography; MS: Mass spectrometry; MSPE: Magnetic solid-phase extraction; RR: Relative recovery; SI-HLLE-DSPE-DLLME-SFO: Salt induced-homogenous liquid-liquid extraction, dispersive solid phase extraction, and dispersive liquid–liquid microextraction based on the solidification of floating organic droplet; SPE: Solid-phase extraction; Ultraviolet.

## Data Availability

The data supporting reported results can be found in the laboratory databases of the Faculty of Sciences of the University of Lisbon.

## Sample Availability

Samples of the compounds studied in the present work are available from the authors at reasonable request.
